# Effect of Voltage Measurement on the Quantitative Identification of Transverse Cracks by Electrical Measurements

**DOI:** 10.3390/s16040427

**Published:** 2016-03-24

**Authors:** Lakshmi Selvakumaran, Gilles Lubineau

**Affiliations:** COHMAS Laboratory, Physical Science and Engineering Division, King Abdullah University of Science and Technology (KAUST), Thuwal 23955-6900, Saudi Arabia; Lakshmi.selvakumaran@kaust.edu.sa

**Keywords:** transverse cracking, electrical tomography, sensitivity, laminated composites

## Abstract

Electrical tomography can be used as a structural health monitoring technique to identify different damage mechanisms in composite laminates. Previous work has established the link between transverse cracking density and mesoscale conductivity of the ply. Through the mesoscale relationship, the conductivity obtained from electrical tomography can be used as a measure of the transverse cracking density. Interpretation of this measure will be accurate provided the assumptions made during homogenization are valid. One main assumption of mesoscale homogenization is that the electric field is in the plane. Here, we test the validity of this assumption for laminates with varying anisotropy ratios and for different distances between the cracked ply and surface that is instrumented with electrodes. We also show the equivalence in electrical response between measurements from cracked laminates and their equivalent mesoscale counterparts. Finally, we propose some general guidelines on the measurement strategy for maximizing the accuracy of transverse cracks identification.

## 1. Introduction

Composite laminates used in structural applications can be degraded by multiple damage mechanisms, which are barely visible until failure. Engineers then need non-destructive testing (NDT) methods to identify the damage mechanisms during service. However, most NDT techniques require a regular maintenance schedule which involves operational shut-down, costly and bulky equipment, and skilled man power. This incurs time and money. To minimize this, structural health monitoring (SHM) techniques have been developed.

SHM aims to use embedded sensors and actuators on the structure to progressively monitor parameters that can provide an indirect measure of the health of the structure. Some of the SHM techniques are acoustic emission [1], modal analysis [2], eddy current analysis [3], *etc*. Most of these techniques cannot be applied in *real-time* thereby limiting the application. Electrical tomography (ET) is a commonly used medical imaging technique that is gaining popularity as a SHM technique, as it can be used *in-situ* and in *real-time*. In ET, electrical current is applied between a pair of electrodes and voltage is measured between the subsequent pair of electrodes. The measurements are then used to reconstruct the conductivity of the object by solving an inverse problem. Provided that the damage causes significant change in the conductivity of the laminate, the reconstructed conductivity can be interpreted in terms of the damage mechanisms. As the process is ill-posed, we need regularization methods to obtain the conductivity and numerical models to interpret the conductivity [4,5].

We developed a mesoscale model that can effectively describe the effect of transverse cracks and local delamination through damage indicators that modify the conductivity of the damaged ply. The mesoscale model helps in regularizing the inverse problem and also provides a means to interpret the conductivity in transverse cracks and local delamination. Homogenizing at the meso scale mainly consists of substituting the micromechanical description of the degraded ply (in which cracks are explicitly represented) with an equivalent homogeneous but damaged representation. Further studies showed how the material and geometric properties of the laminate influence the sensitivity of the measurements towards transverse cracks. In this work, we discuss an optimal measurement strategy that will maximize the amount of information we could obtain without violating the mesoscale homogenization assumptions, thereby minimizing the approximation error. In an ET system, when the electrodes are placed on the surface of the laminate, the distance between the current injection electrodes is the distance through which the current travels. Hence the potential measurements obtained at the electrodes located between these current injection electrodes, carry vital information about the changes in conductivity along the path. Identification of transverse cracks is possible due to the mesoscale relationship obtained through homogenization which links the mesoscale conductivity to the transverse cracking density.

For the mesoscale conductivity to be an accurate representation of the crack density, the path over which the voltage difference is measured should have in-plane electric field. This is because mesoscale homogenization was carried out with the assumption of in-plane electric field. However, the length over which the electric field remains in the plane can be affected by the level of anisotropy within the laminate and also the depth of the lamina with respect to the thickness of the laminate. The effects of these two factors are studied.

The objective of the current paper is thus to propose guidelines on the voltage measurement strategy such that the transverse crack density can be best identified. We then precisely define the consequence of mesoscale modeling choices, explained in our previous publications, on practical configurations. We begin by reviewing the results of mesoscale homogenization of transverse cracks followed by the description of the quasi-static conduction model for electrical tomography. Then the study on the effect of anisotropic ratio and depth of the ply within the laminate on the validity of mesoscale assumption is presented and discussed.

## 2. Transverse Cracking in Laminates and Its Representation in the Mesoscale

The transverse cracks are large cracks that spans through the whole thickness of the ply. They establish a quasi periodical network along the transverse direction; the density of cracks can be quantified by a dimensionless cracking density which is given by:

(1) $$\rho =\frac{H}{L}$$

where *H* is the thickness of the ply and *L* is the average spacing between the two consecutive cracks. As the transverse cracks multiply, *ρ* increases. Experiments show that *ρ* saturates at around 0.8–1.2 [6,7], beyond which local delaminations at the tips of the transverse cracks starts to develop.

**Figure 1:**
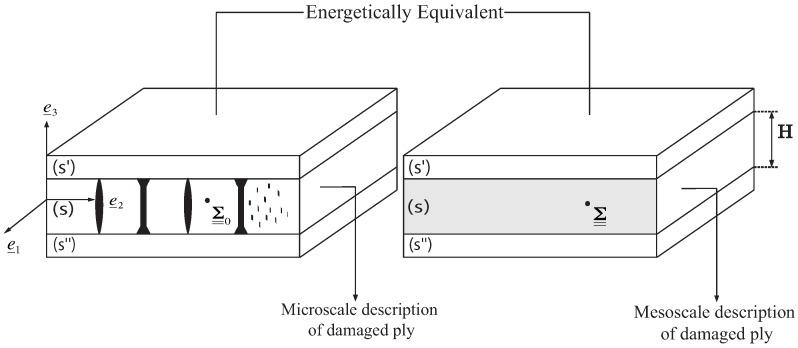
Micro-meso equivalence.

It is of course irrelevant, both for integrity prediction or for integrity monitoring, to account for each individual transverse crack. Doing so would be computationally intractable as well as very dependent on variabilities that are inherent to composite structures at the micro-level. One solution is to use instead a smeared representation such as mesoscale homogenization. By micro-meso homogenization, the transverse cracks are accounted for through their influence on modified properties at the mesoscale, such as the stiffness or conductivity tensor of the ply. The main features of mesoscale homogenization are: (1) it provides a continuous representation of discrete damage through damage indicators and (2) the damage indicators are intrinsic (*i.e.*, they directly quantify the degradation state of the ply but do not depend on the lamination parameters such as orientation or thickness). The electrical homogenization is performed in such a way that equivalenece in electrical energy is ensured between both scales (see Figure 1). [8,9] details the procedure of electrical homogenization for transverse cracking and local delamination.

Through mesoscale homogenization, it has been shown that transverse cracks modify the transverse conductivity of the laminate and the conductivity tensor for a composite ply that has been damaged has the form

(2) $$\underline{\underline{\Sigma }}={\left[\begin{array}{ccc} \Sigma_{11}^{0} & 0 & 0\\ 0 & \Sigma_{22}^{0}\left(1-d\right) & 0\\ 0 & 0 & \Sigma_{33}^{0} \end{array}\right]}_{\left({\underline{e}}_{1},{\underline{e}}_{2},{\underline{e}}_{3}\right)}$$

where *d* denotes the damage due to transverse cracks. Explicit relationship between the damage indicator *d* and transverse cracking density can be obtained for a given conductivity and ply geometry through the procedure outlined in [8,9].

## 3. Electrical Tomography

**Figure 2:**
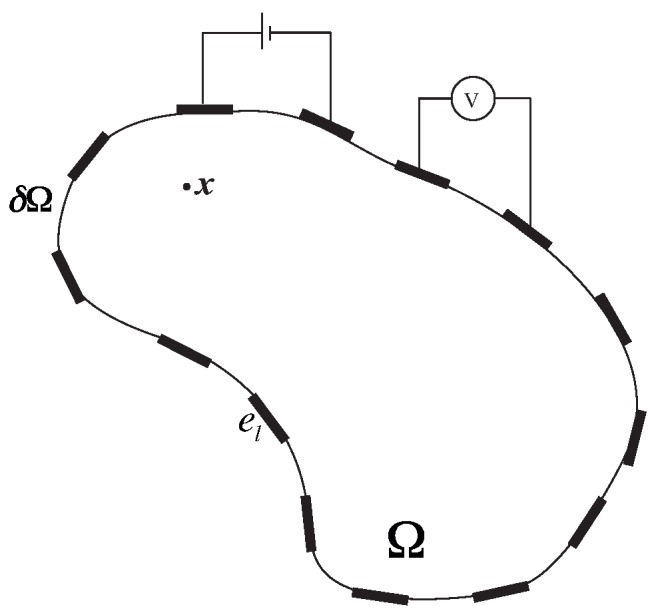
Schematic of electrical tomography.

The experimental configuration of electrical tomography can be equivalently represented by the set of equations described below. Let Ω be a domain with electrodes $el=1,2,...,n_{el}$ on the surface δΩ. Let $\underline{\underline{\Sigma }}$ denote the electrical conductivity of the bulk material Ω and $z_{el}$ denote the contact impedance between the electrode el and δΩ. In electrical tomography, current is applied between pairs of electrodes and voltage is then measured between the other pairs of electrodes. Let $I_{el}$ be the current applied at the surface of the electrodes used for current injection, $S_{e_{l}}$, and $\left\{V\right\}=\left\{V_{1},...,V_{n_{el}-2}\right\}$ denote the set of voltage measurements obtained from the electrodes other than the injection electrodes. The schematic of the system is described in Figure 2. We denote the electric potential, electric field and current density at any point, $\underline{x}$, within the domain Ω as $\left(u,\underline{E},\underline{J}\right)$ respectively. We solve the quasi-static conduction problem given as: to find *u* such that,

- *Kinematic admissibility*
  (3) $$\underline{E}=-\nabla u\;\forall \underline{x}\in \;\Omega$$
- *Static admissibility*
  (4) $$\begin{array}{c} \nabla .\underline{J}=0\;\forall \underline{x}\in \;\Omega \end{array}$$
  (5) $$\begin{array}{c} \int_{e_{l}}\underline{J}.\underline{n}dS_{e_{l}}=I_{el}\;on\;S_{e_{l}},e_{l}=\text{Inj.}\;\text{elec.}\;1 \end{array}$$
  (6) $$\begin{array}{c} \int_{e_{l}}\underline{J}.\underline{n}dS_{e_{l}}=-I_{el}\;on\;S_{e_{l}},e_{l}=\text{Inj.}\;\text{elec.}\;2 \end{array}$$
  (7) $$\begin{array}{c} \underline{J}.\underline{n}=0\;on\;\delta \Omega \backslash \cup \;e_{l} \end{array}$$
- *Constitutive equations*
  (8) $$\begin{array}{c} \underline{J}=-\underline{\underline{\Sigma }}⊗\underline{E}\;\forall \underline{x}\in \;\Omega \end{array}$$
  (9) $$\begin{array}{c} V_{el}-u=z_{el}\left(\underline{J}.\underline{n}\right)\;on\;e_{l},l=1,...,n_{el}-2 \end{array}$$
- *Conservation equations*
  (10) $$\begin{array}{c} \sum_{el=1}^{n_{el}}I_{el}=0 \end{array}$$
  (11) $$\begin{array}{c} \sum_{el=1}^{n_{el}}V_{el}=0 \end{array}$$

where $z_{el}$ denotes the contact impedance of the electrodes. In the studies carried out in the work, $z_{el}$ is taken to be 0.01 Ω.m and $I_{el}$ is taken as 25 mA. The problem is then solved using COMSOL Multiphysics using linear elements of size 0.1 mm × 0.1 mm × 0.1 mm. Once the problem is solved, the average surface voltage at each electrode is obtained and is taken to be the measurements.

## 4. Optimal Measurement Strategy

In Section 2, it has been shown that the effect of transverse cracking density, *ρ* can be equivalently represented at the mesoscale as a modification of the transverse conductivity of the ply. The accuracy of the equivalence however depends on whether the primary assumptions for mesoscale homogenization are met. A main assumption in the mesoscale homogenization is that the electric field is in-plane. In typical ET systems for laminates, current is passed through the electrodes that are placed at regular intervals on the surface of the laminate. Then it is essential to identify a suitable configuration in which the electrical measurements are obtained over the area that has in-plane electric field. Here, we study two factors that affect the length over which the electric field remains in-plane, namely, anisotropy ratio *λ* (ratio of conductivity in fiber direction to the conductivity in the transverse direction) and depth of the cracked ply within the laminate.

### 4.1. In-Plane Ratio for Various Levels of Anisotropy

The current injection electrodes should be placed far enough from each other to provide an in-plane electric field. For a given distance between the current injection electrodes, the larger the area of in-plane electric field, the more measurement electrodes can be placed. This zone can be identified by calculating the ratio of the in-plane to the total energy at any local point given by the expression:

(12) $$\begin{array}{c} \text{In-plane}\;\text{ratio}=\frac{E_{x}.\Sigma_{x}.E_{x}+E_{y}.\Sigma_{y}.E_{y}}{\underline{E}.\underline{\underline{\Sigma }}.\underline{E}} \end{array}$$

If the ratio is 1, then the field is completely in-plane. The factors which can influence this parameter are (i) anisotropy ratio (the ratio of the conductivity in the fiber direction to the conductivity in the transverse direction); (ii) width of the injection electrodes; (iii) width of the measurement electrodes. We will study the influence of each of these parameters on the in-plane to total energy ratio.

**Figure 3:**
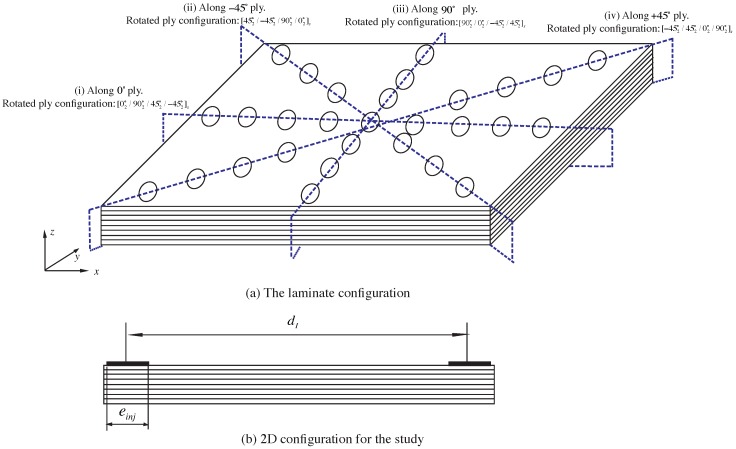
The electrodes configuration: (**a**) Laminate set-up and (**b**) the 2D study configuration.

To generalize the study, we consider a Quasi-isotropic laminate ${\left[0_{2}^{\circ }/90_{2}^{\circ }/45_{2}^{\circ }/-45_{2}^{\circ }\right]}_{s}$ with an array of electrodes along each principal direction of the plies as shown in Figure 3a. In this study, the electrodes are considered to be on only one side of the laminate considering that in most applications only one side is accessible due to logistical or safety reasons. Quasi-isotropic laminate is chosen since it is one of the most commonly used laminate configuration and since the results obtained from Quasi-isotropic laminate can be easily translated to cross-ply laminates.

The thickness of each ply is 0.25 mm. Each array of electrodes along each direction will be used to evaluate the presence of transverse cracks in a specific ply. This is the ply whose fiber direction is perpendicular to the direction of the electrodes. To make it easier to understand, we refer to each of these cross-section using a rotated basis with ${\underline{e}}_{1}$ pointing along the direction of the electrodes. For each cross-section marked as (i), (ii), (iii) and (iv) in Figure 3a, the location of the ply whose matrix cracks can be evaluated varies (For e.g., for cross-section (i), the local $90^{\circ }$ plies are the second and seventh ply from the top).

**Figure 4:**
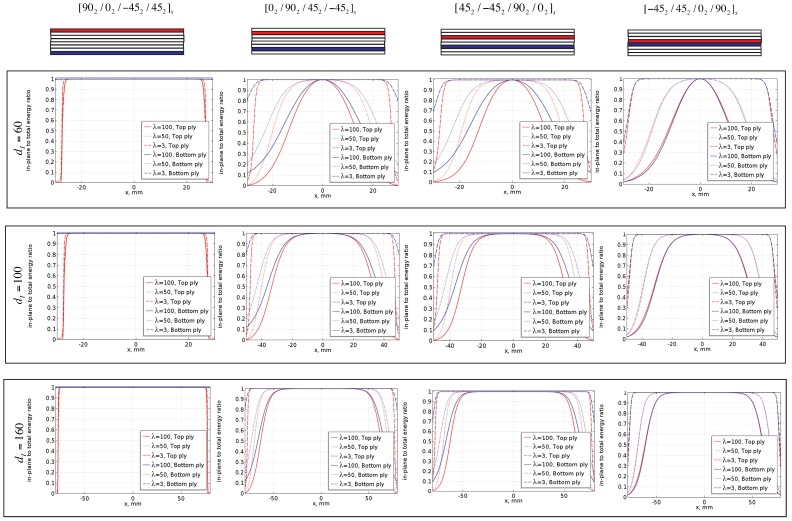
Effect on anisotropic ratio on the in-plane ratio.

Now, we vary the distance between the two current carrying electrodes as $d_{I}=\left[160,100,60\right]$ mm for each of these cross-sections and the ratio of the in-plane energy to the total energy along the local $90^{\circ }$ plies (both the plies above (referred as top ply) and below the mid-plane (referred as bottom ply)) is plotted. The maximum length ($d_{in-plane}$) for which this ratio is above 0.99 is identified for each cross-section and the ratio of $d_{in-plane}/d_{I}$ is plotted. To understand the effect of the anisotropic ratio, *λ*, we take three different ratios (100, 50 and 3). Anisotropy ratios of 50 and 100 represent the typical ratio observed in CFRP laminates. Anisotropy ratio of 3 is chosen considering that better sensitivity towards transverse cracks can be achieved for lower anisotropy ratio and that nano-doped GFRP have been shown to have anisotropy ratio in the range of 3. The width of the injection electrodes are taken as 5 mm. The results are shown in Figure 4.

**Figure 5:**
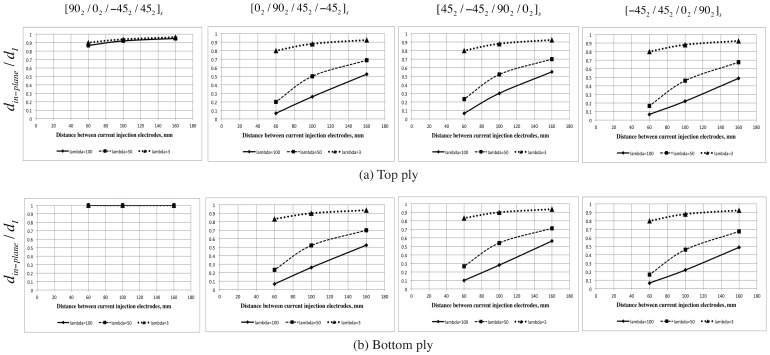
Relationship between $\frac{d_{in-plane}}{d_{I}}$, the distance between the current injection electrodes and the anisotropy ratio *λ*. We detail here in (**a**) the results for the top 90 degree ply and in (**b**) the results for the bottom 90 degree ply.

From Figure 4, we observe the following:

1. The higher the anisotropic ratio, the lower the distance over which the electric-field remains in-plane. For λ=3, the electric field is in-plane through most distance between the current injection electrodes for all the different locations of the $90^{\circ }$ plies. However, for λ=100 and 50, the in-plane ratio is good only when the $90^{\circ }$ ply is at the top or the bottom but otherwise very poor. The immediate consequence is that the length over which the mesoscale assumption is valid drastically reduces with increasing anisotropy.
2. Given that meaningful information can be extracted only from the measurements obtained in-between the current injection electrodes, anisotropy primarily influences the number of measurements that can be obtained within a given distance between the current injection electrodes. This in turn will influence the resolution of the conductivity map that can be reconstructed. This further shows that decreasing the anisotropic ratio by introducing conductive nano particles improves not only the sensitivity towards transverse cracks [10] but also helps in obtaining more measurements within a given area.
3. We observe an almost linear relationship between the $d_{I}$ and $d_{In-plane}$ for all the four configurations as seen in Figure 5. Thus, given the anisotropy ratio and the location of the $90^{\circ }$ plies, we can apriori determine the distance over which measurements must be obtained such that mesoscale homogenization is valid.

### 4.2. Effect of Depth on Mesoscale Approximation

Next, we study the equivalence between the measurements from the mesoscale model and the cracked model (micro) and compare these quantities from the plies at different depths of the composite laminate. This study is carried out because most laminates in classical industrial applications are as thick as 10 mm. However, studies carried out so far do not account for the effect of thickness on mesoscale homogenization. The objective is to understand the effect of depth of the cracked ply from the plane of the electrodes on the equivalence between the measurements. Considering that laminates with high anisotropy ratio are not suitable to track transverse cracking, we limit this study to a laminate with anisotropy ratio of 3.

**Figure 6:**
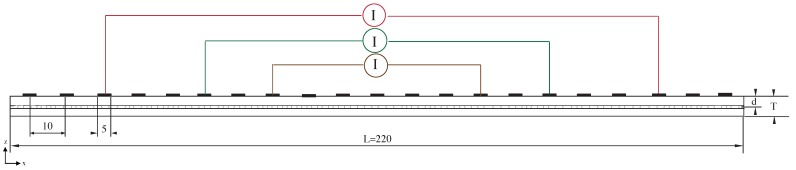
Geometrical configuration and different injection lengths considered.

We consider a laminate of length L=220 mm and thickness T=10 mm. The plies surrounding the cracked $90^{\circ }$ ply are considered as homogenized and exhibiting quasi-isotropic properties. Hence, the laminate configuration considered is denoted as: [quasi-iso/$90^{\circ }$/quasi-iso]. The thickness of the $90^{\circ }$ ply is taken as 1 mm. An array of electrode is taken on the top surface of the laminate with electrodes spaced at 10 mm apart from each other. We define depth ratio as the ratio of the depth of $90^{\circ }$ ply from electrode plane to total thickness of the laminate and is varied as d/T=[0.15,0.25,0.45,0.55,0.85]. Current is injected between electrodes that are at a distance of $d_{I}=\left[160,100,60\right]$. The considered geometry and the different injection lengths are shown in Figure 6. The $90^{\circ }$ is considered to be cracked with a uniform cracking density of ρ=0.5. We equivalently represent the effect of the cracks on a laminate with mesoscale conductivity. Measurements are obtained in the electrodes between the current injection electrodes for both the cracked and equivalent mesoscale laminate.

Two quantities of interest namely, *relative error (%)* and *relative variation (%)* is plotted for all the measurements. *Relative error* is defined as the error due to mesoscale approximation and is given a as:

(13) $$\mathrm{Relative error}=\frac{V_{meso}-V_{micro}}{V_{micro}}\times 100\%$$

*Relative variation* is defined as the ratio of the approximation error to the variation due to the introduction of cracks and is given as:

(14) $$\mathrm{Relative variation}=\frac{V_{meso}-V_{micro}}{V_{nocracks}-V_{micro}}\times 100\%$$

**Figure 7:**
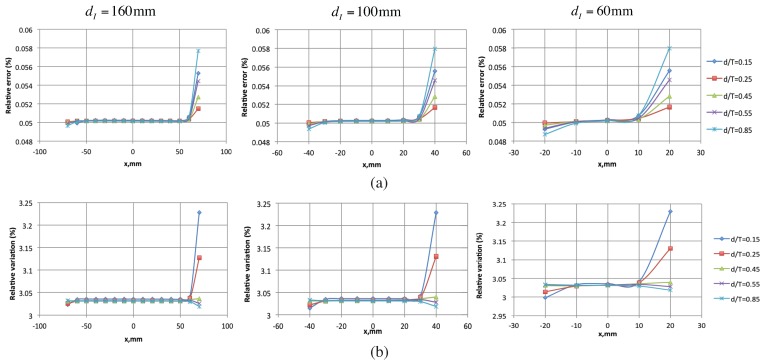
(**a**) Relative error and (**b**) relative variation of voltage measurements for different depths of the cracked ply.

**Figure 8:**
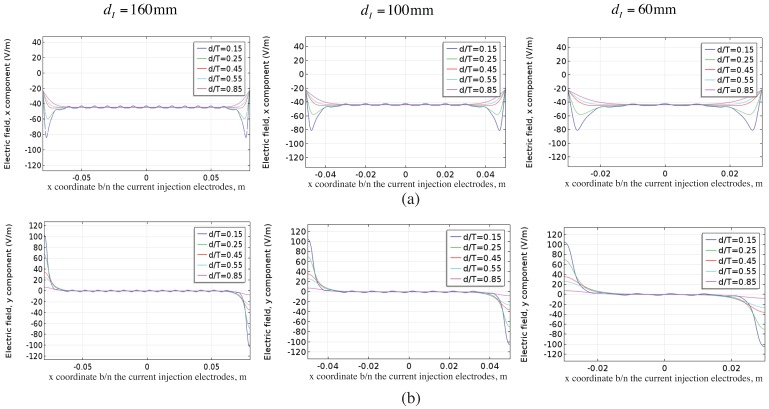
Electric field in (**a**) x direction and (**b**) y direction along the cracked ply at different depths within the laminate.

Figure 7 shows these two quantities plotted as a function of the location of the electrodes for the different depth ratios. Based on the results, we see that, for all the depths considered,

1. The magnitude of *% relative error* and *% relative variation* is very small (0.05% and 3.3% respectively) showing that mesoscale homogenization is an valid representation of the effect of transverse cracks within the laminate.
2. The *% relative error* and *% relative variation* is uniform at the electrodes that are more than 20mm away from the current injection electrodes. The electrodes closer to the current injection electrodes show deviations which can be correlated to the perturbation in the in-plane electric field response around the injection electrodes. This is evident from the electric field profile plotted in Figure 8. The violation in the homogenization assumption close to the injection electrodes results in the resulting perturbation. Although smaller in magnitude, the perturbations can impact the quality of the reconstruction and is best neglected. Hence, it is proposed that only the measurements from electrodes that are more than 10mm away from the injection electrodes be used for the reconstruction.

## 5. Discussion

Based on the above results, it can be seen that,

1. Anisotropy ratio strongly influences the distance between which the electric field remains in-plane. Previous studies have shown that laminates with high anisotropy ratio show very low sensitivity towards transverse cracks. Based on this, we reiterate the importance of improving the electrical properties by introducing nano particles in the laminate matrix if electrical tomography is to be used as a viable structural health monitoring technique. However, adding nanoparticles to a polymer composite can have an effect on the mechanical properties. In our previous review [11], we concluded that adding nanoparticles has to be done with care. Large improvement in the mechanical properties cannot be expected on a regular basis. For some techniques, a decrease in some mechanical properties might even be observed. It is important to introduce the particles with well-chosen techniques and to introduce as few particles as possible to ensure the new electrical functionality without compromising the mechanical integrity.
2. Depending on the location of the $90^{\circ }$ ply and the length between the injection electrodes, the distance over which the electrodes can be placed can be determined from Figure 5 for various anisotropy ratios.
3. For laminates with low anisotropy (less than 3) and thickness of up to 10mm, the electric field remains in-plane and uniform 20 mm away from the injection electrodes irrespective of the position of the cracked ply within the laminate. *% Relative error* and *% relative variation* is very low in magnitude within this length.
4. For laminates with low anisotropy, transverse cracks can be tracked with high resolution and can be quantified accurately using the mesoscale relationship obtained from the homogenization procedure.

## 6. Conclusions

We have studied the influence of the anistropy ratio of the material and the depth of the probing ply within the laminate on the validity of the mesoscale assumption and hence on the accuracy of the mesoscale approximation.

Depending on the anisotropy ratio of the material, the out-of-plane component (with respect to the plane of the laminate) of the electrical field in the bulk material can be more or less important. In more isotropic materials, the in-plane component is more important, ensuring that we can safely use the mesoscale homogenization framework.

In terms of a general guideline, the number and quality of measurements can be maximized by lowering the anisotropic ratio. With the objective of maximizing the number of data points (electrodes) without violating the mesoscale framework, we found that the minimum distance between the voltage measurement and the current injection electrodes decreased with the anisotropy ratio. Decreasing the anisotropy ratio therefore is a good choice because more data points can be collected. In a relatively isotropic material, this minimum distance would be around 20 mm.

The effect of highly anisotropic electrical behavior on the quality of the data obtained during electrical monitoring of composite structures is generally not studied. We show here that the level of electrical anisotropy should be carefully considered when designing an electrical tomography inspection technique. The placement of the electrodes should be carefully adapted depending on the anisotropy ratio as it might strongly affect the quality of the measured data. Integrated, *in-situ* and real-time electrical monitoring systems of composites are promising for structural health monitoring, but should be carefully designed to account for the very specific features of such materials.
